# The CXCR4 antagonist plerixafor enhances the effect of rituximab in diffuse large B-cell lymphoma cell lines

**DOI:** 10.1186/s40364-016-0067-2

**Published:** 2016-06-14

**Authors:** Linn Reinholdt, Maria Bach Laursen, Alexander Schmitz, Julie Støve Bødker, Lasse Hjort Jakobsen, Martin Bøgsted, Hans Erik Johnsen, Karen Dybkær

**Affiliations:** Department of Haematology, Aalborg University Hospital, Sdr Skovvej 15, Aalborg, DK-9000 Denmark; Clinical Cancer Research Center, Aalborg University, Sdr Skovvej 15, Aalborg, DK-9000 Denmark; Department of Clinical Medicine, Aalborg University, Sdr Skovvej 15, Aalborg, DK-9000 Denmark

**Keywords:** Diffuse large B-cell lymphoma, DLBCL, CXCR4, CXCR4 antagonist, Plerixafor, AMD3100, Rituximab, Anti-CD20 antibody, Drug combination study, in vitro

## Abstract

**Background:**

Diffuse large B-cell lymphoma (DLBCL) is an aggressive disease with variable clinical outcome, accounting for at least 25-30 % of adult non-Hodgkin lymphomas. Approximately one third of DLBCL patients are not cured by the currently used treatment regimen, R-CHOP. Hence, new treatment strategies are needed. Antagonizing the CXCR4 receptor might be promising since the CXCR4-CXCL12 axis is implicated in several aspects of tumor pathogenesis as well as in protection from chemotherapeutic response. In Burkitt lymphoma, the CXCR4 antagonist plerixafor has already been shown to enhance the therapeutic effect of rituximab, the immunotherapeutic agent of R-CHOP; but this is yet to be confirmed for DLBCL. We, therefore, investigated the effect of plerixafor on DLBCL cellular response to rituximab.

**Methods:**

In this in vitro study, human DLBCL cell lines were treated with rituximab and/or plerixafor, concomitantly or in sequence. The trypan blue exclusion method and MTS-based assays were used to evaluate cellular proliferation, whereas flow cytometry was used for assessment of apoptosis status and CXCR4 surface expression level. Linear mixed effects models were used to assess statistical significance.

**Results:**

We observed that simultaneous addition of plerixafor and rituximab resulted in a significant decrease in DLBCL cellular proliferation, compared to monotherapeutic response. The effect was dose-dependent, and concomitant administration was observed to be superior to sequential drug administration. Accordingly, the fraction of apoptotic/dead cells significantly increased following addition of plerixafor to rituximab treatment. Furthermore, exposure of DLBCL cells to plerixafor resulted in a significant decrease in CXCR4 fluorescence intensity.

**Conclusions:**

Based on our results, implying that the anti-proliferative/pro-apoptotic effect of rituximab on DLBCL cells can be synergistically enhanced by the CXCR4 antagonist plerixafor, addition of plerixafor to the R-CHOP regimen can be suggested to improve treatment outcome for DLBCL patients.

**Electronic supplementary material:**

The online version of this article (doi:10.1186/s40364-016-0067-2) contains supplementary material, which is available to authorized users.

## Background

Lymphomas are a class of hematological cancers which can be categorized as either Hodgkin lymphomas or non-Hodgkin lymphomas. Non-Hodgkin lymphomas are the most frequent of the two, with the aggressive diffuse large B-cell lymphoma (DLBCL) accounting for at least 25-30 % of adult non-Hodgkin lymphomas [1].

DLBCL is a clinically, morphologically, and molecularly heterogeneous disease with an unknown etiology [2]. The treatment currently used for DLBCL is a multi-agent regimen combining the anti-CD20 monoclonal antibody rituximab with three chemotherapeutic drugs, i.e. cyclophosphamide, doxorubicin, and vincristine, and with the corticosteroid prednisone (R-CHOP). Addition of rituximab to the treatment regimen of DLBCL patients has improved treatment outcome significantly [3–6]. Even so, 30-40 % of DLBCL patients have refractory disease or relapse after treatment with R-CHOP [7]. Relapsed patients are generally treated with high-dose chemotherapy in combination with autologous stem cell transplantation. The majority of these patients are, however, not eligible for this treatment strategy because of their age, comorbidities, or refractory disease. Thus, this subset of patients is not cured and, consequently, other therapeutic approaches are required.[8]

Rituximab is a genetically engineered chimeric monoclonal antibody consisting of a human Fc region and murine variable regions, recognizing CD20 cell surface molecules abundantly present on most normal and malignant B-cells [9]. Uncertainty remains on how rituximab exerts its therapeutic effects. Several mechanisms have been proposed, including complement-dependent cytotoxicity (CDC), antibody-dependent cellular cytotoxicity (ADCC), and apoptosis. Interestingly, the effect of rituximab was enhanced by the C-X-C chemokine receptor type 4 (CXCR4) antagonist plerixafor (AMD3100) in a related type of lymphoma (Burkitt lymphoma) [10, 11]. This CXCR4 antagonist displays a low-risk safety profile and has already been approved by the U.S Food and Drug Administration (FDA) for clinical use in non-Hodgkin lymphoma patients in combination with granulocyte colony-stimulating factor (G-CSF) for the purpose of mobilizing hematopoietic stem cells [12]. Plerixafor is a specific small molecule CXCR4 inhibitor. More specifically, plerixafor is a bicyclic reversible inhibitor that blocks binding of the CXCR4 receptor ligand, C-X-C chemokine ligand type 12 (CXCL12 or SDF-1α), by binding to an extracellular binding pocket of CXCR4 [13].

CXCR4 is a cell surface receptor implicated in B-cell lymphopoiesis, as demonstrated by severely impaired B-cell lymphopoiesis in CXCR4-deficient mice [14, 15]. It is expressed on normal and many malignant hematopoietic cells; including those of non-Hodgkin lymphomas, multiple myeloma, acute lymphoblastic leukemia (ALL), and chronic lymphocytic leukemia (CLL) [16, 17]. Recently, CXCR4 overexpression was reported to be associated with decreased survival of mice intravenously injected with DLBCL cells [18] and, importantly, with poor prognosis in a cohort of 94 DLBCL patients treated with rituximab-containing regimens [18] and a training/validation cohort of 468/275 DLBCL patients treated with the R-CHOP regimen [19].

The CXCR4-CXCL12 axis appears to be implicated in several biological functions linked to tumor pathogenesis. Proliferative and pro-survival signaling pathways can be induced by activation of CXCR4 on the surface of tumor cells, and blocking the CXCR4 receptor, accordingly, results in inhibition of tumor proliferation [11, 20]. Migration of CXCR4-positive hematopoietic cells is promoted by a gradient generated through stromal cell production of CXCL12 [16]. In line with this, blocking CXCR4 results in inhibition of tumor metastasis [21].

Chemotherapy protection of malignant hematopoietic cells is shown to be induced by interaction with surrounding stromal cells in e.g. the bone marrow. Accordingly, this interaction is suggested to be involved in treatment resistance and persistence of minimal residual disease.[22] By inhibiting the interaction of CXCR4 with its ligand CXCL12, several studies have reported that the tumor-promoting signals of stromal cells can be reversed, resulting in more chemotherapy-susceptible tumor cells and an increase in the spontaneous apoptosis rate [23–26]. Consequently, blocking the CXCR4-CXCL12 axis might be a promising approach for potentiating the effects of the currently used treatment regimen in DLBCL.

To the best of our knowledge, the effect of combining the CXCR4 antagonist plerixafor with the monoclonal anti-CD20 antibody rituximab has never been evaluated in DLBCL; neither *in vitro*, nor *in vivo*. In this study, we evaluated the *in vitro* effect of combining plerixafor and rituximab, by comparing the level of growth inhibition induced by single agent and combination treatment of DLBCL cell lines. Flow cytometry-based assays were applied to DLBCL cell lines to investigate the combined and solitary effect of the drugs on CXCR4 surface expression and on apoptosis stage. Thus, this study investigates how rituximab and/or plerixafor influence CXCR4 expression, and how the expression of CXCR4 influences drug effect *in vitro*.

## Methods

### Cell line characteristics

In this study, two human DLBCL-derived malignant suspension cell lines were used; RIVA and FARAGE. Both cell lines were kindly provided by Dr. Jose A. Martinez-Climent (Molecular Oncology Laboratory, University of Navarra, Pamplona, Spain). Through systematic dose-response experiments, thoroughly described previously [27], the 50 % growth inhibition (*GI*_50_^G^)-values of the cell lines with respect to rituximab were determined to be 3.3 μg/mL and 27.8 μg/mL, respectively (Additional file 1), demonstrating RIVA to be more sensitive than FARAGE. This difference in rituximab sensitivity was our incentive to proceed with these cell lines. In regards to molecular subtype, RIVA is ABC-like whereas FARAGE is classified as GCB [28]. According to the Leibniz Institute DSMZ-German Collection of Microorganisms and Cell Cultures catalogue (ACC 585), RIVA displays a complex karyotype, carrying i.a. *MYC* rearrangement (t(4;8)(q22;q24)) and *BCL2* amplification (der(18)amp(18)(q21)dup(18)(q21q23)). According to the American Type Culture Collection (ATCC CRL-2630), FARAGE has a more simple karyotype, with trisomy of chromosome 11 as the only listed karyotypic aberration.

### Cell culturing

Cells were maintained in RPMI 1640 medium (Life Technologies, Copenhagen, DK) supplemented with 10 % heat-inactivated fetal bovine serum (Invitrogen, Copenhagen, DK), 100 U/mL penicillin, and 100 μg/mL streptomycin (Life Technologies, Copenhagen, DK), at 37 °C and 5 % CO_2_ in a humidified atmosphere. Cells were passaged regularly to ensure optimal cell growth, and maintained for a maximum of 25 passages to minimize any long-term culturing effects. To ensure that cells were harvested in their exponential growth phase when conducting experiments, cells were incubated at 37 °C and 5 % CO_2_ in a humidified atmosphere for approximately 24 h after seeding. Importantly, both cell lines were identification-validated and examined for mycoplasma infection at the end of their culturing period, to avoid misinterpretation of the experiments due to cross-contamination/mislabeling or mycoplasma-induced changes of cellular properties, respectively. The EZ-PCR Mycoplasma Test Kit (Biological Industries, Beit HaEmek, IL) was used to test for presence of mycoplasma. For identification validation (barcoding), DNA was extracted using the DNeasy Blood and Tissue Kit (Qiagen, Copenhagen, DK) and multiplex PCR performed using the AmpFlSTR® Identifiler® PCR Amplification Kit (Applied Biosystems, Copenhagen, DK). Capillary electrophoresis was completed and analysis performed using Osiris (http://www.ncbi.nlm.nih.gov/projects/SNP/osiris/). Cell line identity was determined by comparing a selection of 9 short tandem repeats against the Leibniz Institute DSMZ-German Collection of Microorganisms and Cell Cultures database (http://www.dsmz.de/services/services-human-and-animal-cell-lines/online-str-analysis.html). Unless otherwise stated, all reported incubation steps were performed at 37 °C in a humidified atmosphere of 5 % CO_2_.

### Administration of reagents

DLBCL cell lines were exposed to rituximab (MabThera®, Roche, Copenhagen, DK) and/or plerixafor (InSolution^TM^ CXCR4 Antagonist I, AMD3100, Merck Millipore, Copenhagen, DK), in sequence or concomitantly. By combining rituximab and plerixafor, we expected a synergistic therapeutic effect, allowing a dose reduction and, thereby, reducing toxicity while maintaining efficacy and minimizing/delaying induction of drug resistance [29]. A final concentration of 20 % Pooled Human AB Serum (HS) (Novakemi AB, Handen, SE) was added, as a source of complement [30] and CXCL12 [31], in order to enable assessment of rituximab-induced CDC and investigate the impact of CXCR4 antagonism, using the same batch of HS (IPLA-SERAB-13517) for all experiments to avoid batch-induced variation. The end point of drug administration was to measure cellular proliferation, apoptosis, and CXCR4 cell surface expression. All reported concentrations are final concentrations.

### Cell proliferation assays

Drug-induced growth inhibition of RIVA and FARAGE cells was assessed in two ways, 1) through enumeration of living cells using the trypan blue exclusion method, 2) through an MTS-based assay.

For the trypan blue exclusion method, cells were seeded out in 24-well culture plates at a concentration of 0.3 × 10^6^ cells/mL, 24 h before drug/saline and HS was added. Rituximab (10 μg/mL) and plerixafor (500 μM) was added either concomitantly or with a 24 h time lag. Following a drug incubation period of 0 h, 24 h, or 72 h, homogenized single cell suspension and trypan blue (Life Technologies, Copenhagen, DK) was mixed 1:1, with inclusion of three counting replicates per well. Cells were counted directly, using a hemocytometer and a light microscope.

For MTS-based assays, cells were seeded out in 96-well culture plates at a concentration of 0.3 × 10^6^ cells/mL, 24 h before drug/saline was added. A 5-point 2-fold serial dilution was applied in triplicates, each drug alone as well as the two drugs combined (C1 + C1, C2 + C2, and so forth). Rituximab in concentrations ranging from 4.17 μg/mL to 66.67 μg/mL, and plerixafor from 208.33 μM to 3333.33 μM. After drug addition, plates were incubated for 30 min before adding HS. After a 48 h incubation period, the number of metabolically active cells was estimated by adding the MTS-containing CellTiter 96® AQueous One Solution Reagent (Promega, Madison, WI, USA) at a concentration of 20 % of the pre-addition well content, incubating for exactly two hours, shaking for 10 s, and finally measuring absorbance at 492 nm, using an Optima-Fluostar plate reader (BMG LABTECH, Ortenberg, DE). To avoid border effect, all border wells were omitted from data analysis.

### Flow cytometry-based analysis

CXCR4 cell surface expression level and apoptosis status of RIVA and FARAGE cells was assessed concomitantly by flow cytometry-based analysis. 24 h before administration of drug/saline, 600 μL cell suspension per well was seeded out in 24-well culture plates at a concentration of 0.3 × 10^6^ cells/mL. After administering rituximab (10 μg/mL) and/or plerixafor (500 μM), plates were incubated for 30 min before addition of HS. Drug-exposed and untreated cells were harvested and stained following a 48 h incubation period. The PE Annexin V Apoptosis Detection Kit I (BD Biosciences, Copenhagen, DK) was applied as described by the manufacturer, with the following modifications. Cells were only washed once and in stain buffer, after which they were resuspended in 100 μL 1X Binding Buffer. Immediately prior to addition of 7-Amino-Actinomycin (7-AAD) and PE-conjugated Annexin V antibody, 10 μL APC-conjugated anti-CXCR4 antibody (clone 12G5, BD Biosciences, Copenhagen, DK) was added. Following incubation at room temperature in the dark for 15 min, 100 μL 1X Binding Buffer was added to each tube. Unstained cells as well as single staining with the appropriate antibodies were included as controls. A BD FACSCanto^TM^ II (BD Biosciences, Copenhagen, DK) was used for analysis, and data were analyzed using FlowJo Software (Tree Star Inc., OR).

### Statistical analysis

To evaluate significance between treatment groups, linear mixed effects models were applied with experimental replicate as random effect and either number of living cells, fraction of apoptotic/dead cells, or log-transformed CXCR4 expression values as dependent variable. To evaluate the type of drug interaction (i.e. additive, antagonistic, or synergistic), number of living cells was used as dependent variable in a linear mixed effects model with interaction between rituximab and plerixafor as independent variable and experimental replicate as random effect. A significance level of 0.05 was applied, and statistical significances between selected groups are indicated on the figures. The treatment group without significance denotation is the reference. All data analyses were performed using the statistical software R, version 3.2.2.

## Results

### The CXCR4 antagonist plerixafor significantly enhanced the rituximab-induced effect on growth inhibition of diffuse large B-cell lymphoma cell lines

**Fig. 1:**
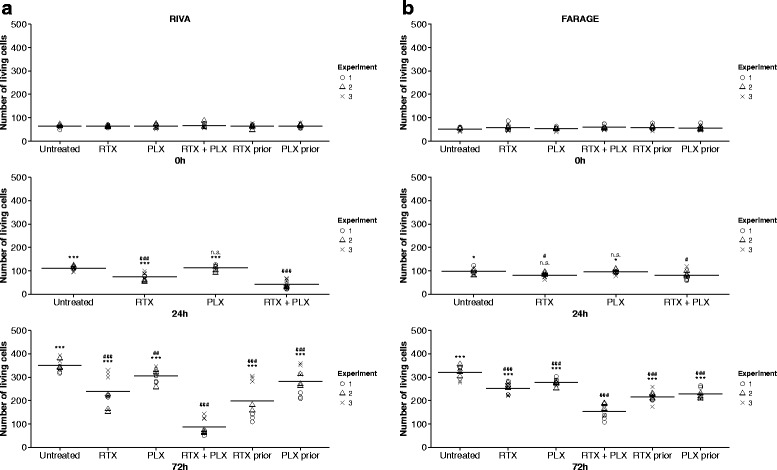
The growth-inhibitory effect of rituximab was significantly enhanced by plerixafor, in a drug sequence-dependent manner. The diffuse large B-cell lymphoma (DLBCL) cell lines RIVA (**a**) and FARAGE (**b**) were exposed to single agent treatment with the anti-CD20 antibody rituximab (10 μg/mL) or the CXCR4 antagonist plerixafor (500 μM) as well as to combination treatment, either concomitantly or with a 24 h time lag. The number of living cells was determined by the trypan blue exclusion method, 0 h, 24 h, and 72 h after drug administration. Horizontal lines represent the mean of three independent experiments, and each geometric symbol represents a counting replicate. *RTX prior* and *PLX prior* are excluded from the 24 h plots since they are equivalent to the *RTX* and *PLX* groups, respectively, at this time point. Statistical significance between groups was determined using a two-level linear mixed effects model. When using concomitant treatment as reference (*RTX + PLX*): *p < 0.05, ***p < 0.001, n.s. no significant difference; when using untreated control (*Untreated*) as reference: ^#^ *p* < 0.05, ^##^ *p* < 0.01, ^###^ *p* < 0.001, n.s. no significant difference; RTX, rituximab; PLX, plerixafor; RTX prior, RTX administered 24 h before PLX; PLX prior, PLX administered 24 h before RTX

To determine if plerixafor modulates the effect of rituximab-induced growth inhibition, RIVA and FARAGE cells were treated with rituximab (10 μg/mL) and/or plerixafor (500 μM) for up to 72 h, and the number of living cells was counted by the trypan blue exclusion method, after 0 h, 24 h, and 72 h of drug exposure. For both cell lines, rituximab single agent treatment resulted in a significant decrease in the number of living cells after 24 h-72 h (*p* < 0.05), and plerixafor monotherapy in a significant decrease after 72 h (*p* < 0.05) (Fig. 1). Remarkably, by administering plerixafor and rituximab concomitantly, a pronounced reduction (*p* < 0.001) in the number of living cells, as compared to both the untreated control and single agent treatments, was observed after 24 h-72 h for RIVA (Fig. 1a) and 72 h for FARAGE (Fig. 1b). To evaluate the type of drug interaction, linear mixed effects models with interaction between rituximab and plerixafor were applied, revealing that a synergistic interaction can be assumed at 24 h-72 h for RIVA (*p* < 0.001) and 72 h for FARAGE (*p* < 0.01).

For investigating if drug combination sequence has impact on outcome, drugs were added sequentially with a 24 h gap, in addition to the single agent treatments and the concomitant administration. For both cell lines, the most pronounced effect was observed following concomitant treatment (Fig. 1). By adding rituximab and plerixafor sequentially, the decrease in the number of living cells was significantly smaller for both cell lines, compared to concomitant exposure for 72 h (*p* < 0.001). Results were especially pronounced for RIVA, the more rituximab-sensitive cell line. Concomitant administration resulted in a 4-fold reduction in the number of living cells as compared to the untreated control, whereas initial administration of rituximab followed by plerixafor 24 h later only caused a 1.8-fold reduction (Fig. 1a). Therefore, we conclude that combination sequence of rituximab and plerixafor is of importance, with concomitant administration being superior to sequential administration. Hence, concomitant drug administration was included in subsequent experiments.

**Fig. 2:**
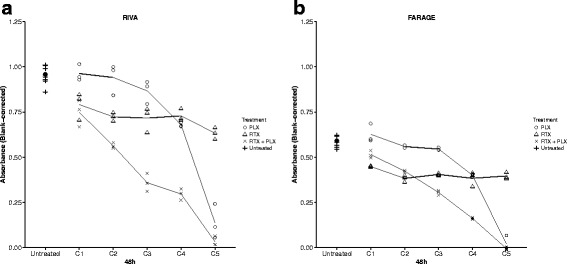
The growth-inhibitory effect of rituximab was remarkably enhanced by plerixafor, in a drug concentration-dependent manner. The diffuse large B-cell lymphoma (DLBCL) cell lines RIVA (**a**) and FARAGE (**b**) were exposed to a two-fold serial dilution of the anti-CD20 antibody rituximab (4.17-66.67 μg/mL) and/or the CXCR4 antagonist plerixafor (208.33-3333.33 μM) for 48 h. When in combination, corresponding concentrations of the drugs were administered (i.e. C1 + C1, C2 + C2, and so forth). Blank-corrected absorbance values were obtained through MTS-based experiments. Data are presented as mean of a minimum of three technical replicates, with each individual replicate presented as a geometric symbol. PLX, plerixafor; RTX, rituximab

MTS-based assays were performed to further investigate the effect of adding plerixafor to rituximab treatment of DLBCL cells. A 5-point 2-fold serial dilution (rituximab: 4.17-66.67 μg/mL; plerixafor: 208.33-3333.33 μM) was applied to RIVA and FARAGE cells, alone as well as in combination, 48 h before addition of an MTS-containing reagent, with absorbance readings at 492 nm 2 h later. For both cell lines, rituximab monotherapy resulted in a reduction in the number of metabolically active cells, as compared to the untreated control, for all concentrations tested (Fig. 2). Plerixafor monotherapy, on the contrary, showed only little to no effect at low concentrations, whereas the reduction in the number of metabolically active cells was very pronounced at the highest concentration tested, as compared to the untreated control (Fig. 2). Whether the effect of high plerixafor concentration is due to general cytotoxicity remains to be tested, however. The drug-induced response pattern was very similar between the two cell lines, with optimal treatment choice depending on the drug concentrations used. For the majority of concentrations (C3-C5) applied to the two cell lines, combined treatment exceeded the effect induced by the corresponding concentration of rituximab (Fig. 2). The effect of single agent treatment with plerixafor was exceeded by combined treatment for all concentrations tested; except for the highest concentration where no evident difference was observed (Fig. 2).

In conclusion, we observed that the growth-inhibitory effect of rituximab can be enhanced by the CXCR4 antagonist plerixafor in DLBCL cell lines, presumably in a synergistic manner, with a more pronounced effect for cells displaying higher rituximab sensitivity. Furthermore, we demonstrated that the effect of combination treatment is dose-dependent as well as drug sequence-dependent, with concomitant administration being superior to sequential administration.

### Combining plerixafor with rituximab significantly increased the fraction of apoptotic/dead diffuse large B-cell lymphoma cells

To explore the mechanism of drug effect in RIVA and FARAGE cells, rituximab (10 μg/mL) and/or plerixafor (500 μM) were applied and apoptosis analysis performed 48 h later by flow cytometry, using 7-AAD in combination with Annexin V to distinguish living cells (7-AAD^−^/Annexin V^−^), early apoptotic cells (7-AAD^−^/Annexin V^+^), and late apoptotic/dead cells (7-AAD^+^/Annexin V^+^) from each other.

**Fig. 3:**
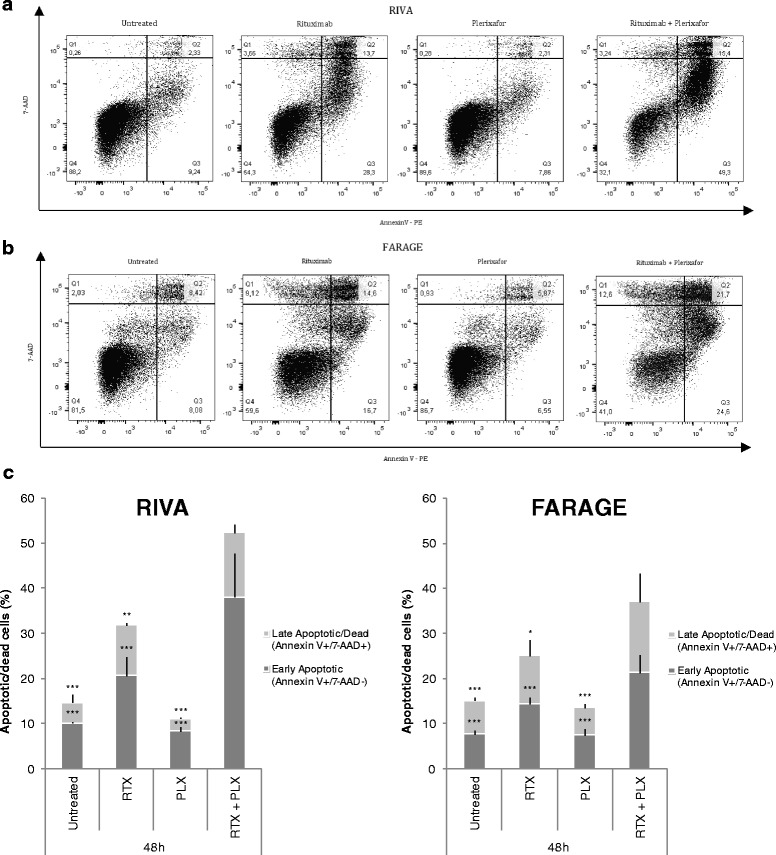
Addition of plerixafor to rituximab treatment significantly increased the fraction of apoptotic cells. The anti-CD20 antibody rituximab (10 μg/mL) and/or the CXCR4 antagonist plerixafor (500 μM) were administered to the diffuse large B-cell lymphoma (DLBCL) cell lines RIVA (**a**) and FARAGE (**b**). The cells were subjected to flow cytometry-based analysis 48 h later, using a combination of 7-AAD and PE-conjugated Annexin V. One representative experiment per cell line is shown, with numbers indicating the percentage of cells in each quadrant. (**c**) The fractions of early apoptotic and late apoptotic/dead cells are summarized as mean of two independent experiments ± SEM, with each experiment containing technical triplicates. Statistical significance between groups was determined using a two-level linear mixed effects model. *p < 0.05; **p < 0.01; ***p < 0.001; RTX, rituximab; PLX, plerixafor; Q1, dead cells (AnnexinV^−^/7-AAD^+^); Q2, late apoptotic/dead cells (AnnexinV^+^/7-AAD^+^); Q3, early apoptotic cells (AnnexinV^+^/7-AAD^−^); Q4, living cells (AnnexinV^−^/7-AAD^−^)

In accordance with the proliferation experiment results, administering plerixafor concomitantly with rituximab significantly increased the fraction of early apoptotic (*p* < 0.001) as well as late apoptotic/dead cells (*p* < 0.05), relative to single agent treatments (Fig. 3), indicating apoptosis to be part of the drug response mechanism. As for the proliferation experiments, the effect of combination treatment on apoptosis status was not as pronounced for the less rituximab-sensitive FARAGE cells (Fig. 3c).

In conclusion, the fraction of DLBCL cells in early as well as late apoptosis was significantly increased when plerixafor was administered simultaneously with rituximab, as compared to single agent treatments.

### Plerixafor induced a significant decrease in CXCR4 fluorescence intensity for diffuse large B-cell lymphoma cell lines

**Fig. 4:**
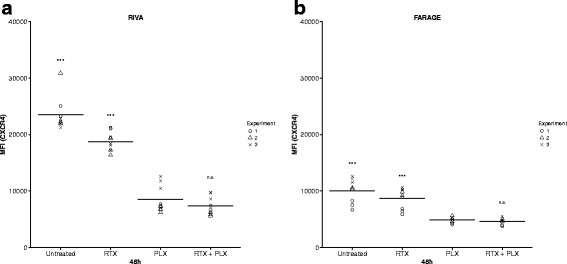
Median CXCR4 fluorescence intensity was significantly decreased following plerixafor exposure. The diffuse large B-cell lymphoma (DLBCL) cell lines RIVA (**a**) and FARAGE (**b**) were exposed to the anti-CD20 antibody rituximab (10 μg/mL) and/or the CXCR4 antagonist plerixafor (500 μM) for 48 h, after which CXCR4 fluorescence intensity was determined by flow cytometry. Horizontal lines represent the mean of three independent experiments, and each geometric symbol represents a technical replicate. Statistical significance between groups was determined using a two-level linear mixed effects model. ***p < 0.001; n.s., no significant difference; MFI, median fluorescence intensity; RTX, rituximab; PLX, plerixafor

Since a post-treatment decrease in bone marrow-expressed CXCR4 was recently reported to be associated with favorable treatment response and significantly better prognosis in a small cohort of non-Hodgkin lymphoma patients [32], we wanted to investigate the effect of plerixafor (500 μM) and/or rituximab (10 μg/mL) on the cell surface expression level of CXCR4. This was conducted 48 h post-drug addition by flow cytometry-based analysis of RIVA and FARAGE cells. A significant decrease in median CXCR4 fluorescence intensity following plerixafor monotherapy was observed for both cell lines, relative to untreated control (*p* < 0.001) (Fig. 4), with the decrease being especially pronounced for the more rituximab-sensitive RIVA cells (Fig. 4a). The plerixafor-induced decrease was not significantly affected by concomitant treatment with rituximab (*p* > 0.05) (Fig. 4).

In conclusion, plerixafor induced a reduction in median CXCR4 fluorescence intensity, which was not significantly altered when rituximab was included in the treatment of DLBCL cells.

## Discussion

CXCR4 is an interesting target in DLBCL since overexpression of this receptor has recently been associated with poor prognosis in a cohort of R-CHOP-treated DLBCL patients [19]. Notably, CXCR4 antagonists can be used as potential drug sensitizers since treatment-induced CXCL12 pathway activation might be involved in acquired drug-resistance mechanisms through several processes, including induction of cancer cell survival, invasion, and stem cell phenotype [33].

In this *in vitro* study on DLBCL cell lines, we investigated the effect of adding the CXCR4 antagonist plerixafor to the anti-CD20 monoclonal antibody rituximab, an immunotherapeutic compound already included in the standard treatment regimen of DLBCL. The effect of plerixafor on the rituximab-induced decrease in the number of proliferating tumor cells was found to be dependent on drug concentration; but for the majority of concentrations tested, plerixafor significantly enhanced the effect of rituximab (Fig. 2). When examining the interaction between rituximab and plerixafor, it was confirmed that a synergistic interaction can be assumed. This might very well be valid *in vivo* as well. Hu *et al.* [11] showed that the therapeutic efficacy of rituximab, as measured by median survival time, was enhanced by plerixafor in a disseminated Burkitt lymphoma model; and although O’Callaghan *et al.* [10] did not find plerixafor to have an effect on survival of Burkitt lymphoma cells *in vitro*, neither when used as single agent treatment nor when in combination with rituximab, they found that combining plerixafor with rituximab *in vivo* resulted in a significant increase in survival of mice with disseminated Burkitt lymphoma, compared to single agent treatment with rituximab, suggesting a substantial clinical effect of combining the two drugs. The much lower dose of plerixafor (10 μM) used by O’Callaghan *et al.* could very likely explain the lack of *in vitro* effect; at low doses we did not observe any effect of plerixafor either (Fig. 2).

The enhancing effect of CXCR4 antagonism on rituximab efficacy is not limited to plerixafor, however. In a recent study by Beider *et al.* [34], addition of the CXCR4 antagonist BKT140 to rituximab treatment was demonstrated to significantly decrease the number of viable lymphoma cells in Burkitt lymphoma cell line studies as well as in the bone marrow of mice with disseminated Burkitt lymphoma xenografts. Another way of targeting CXCR4 is through pepducins, which are cell-penetrating lipopeptide CXCR4 antagonists. O’Callaghan *et al.* [10] observed a significant increase in the rituximab-induced apoptotic effect on Burkitt lymphoma cell lines and primary CLL cells, when combined with the CXCR4 antagonizing pepducins; a treatment strategy which was also capable of increasing the survival of mice with disseminated Burkitt lymphoma xenografts, compared to treating with rituximab alone.

In addition to boosting the effect of rituximab, CXCR4 inhibition has been shown to enhance the effect of other components of the R-CHOP regimen. Lee *et al.* [35] explored the effect of the CXCR4 antagonist T22 on the *in vivo* efficacy of cyclophosphamide in the treatment of established lung metastases from melanoma cells, showing a synergistic drug interaction. In ALL, plerixafor has been shown to enhance vincristine efficacy *in vitro* [36] and *in vivo* [37] and, interestingly, survival of ALL-engrafted mice was extended when plerixafor was added to vincristine treatment [37]. Plerixafor can also sensitize tumor cells to doxorubicin. In an *in vitro* study conducted by Azab *et al.* [23], multiple myeloma cells were demonstrated to be more sensitive to doxorubicin when tumor cell interaction with bone marrow stromal cells was interrupted by plerixafor. These observations suggest that CXCR4 antagonists might exert an even more beneficial effect when combined with the complete R-CHOP regimen, yielding CXCR4 inhibition a promising supplement to the already-implemented R-CHOP treatment in DLBCL.

A phase I clinical trial (NCT00694590) in 24 previously-treated, but relapsed, CLL or small lymphocytic lymphoma (SLL) patients has been conducted with the purpose of investigating if plerixafor sensitizes tumor cells to rituximab-induced killing. The focus was to monitor the safety and efficacy of adding plerixafor to the standard treatment with rituximab, with the primary objective of determining the maximum tolerated plerixafor dose in combination treatment. According to the abstract by Andritsos *et al.* [38], presenting preliminary results for the CLL patients and testing a maximum plerixafor concentration of 0.32 mg/kg, the combined treatment was well-tolerated and, in a proportion of patients, partial remission was observed. Hopefully, this will lead to further clinical examination of plerixafor in combination with R-CHOP.

The primarily used plerixafor concentration in our studies is quite high (500 μM); however, at this concentration, a synergistic effect with rituximab can be assumed whereas single agent plerixafor treatment does not appear to be toxic to the lymphoma cells (Fig. 1). For healthy human volunteers treated with 0.32 mg/kg subcutaneously administered plerixafor, a maximum plasma concentration of 1000 μg/L was estimated (~ approximately 2 μM) [39]. For comparison, a dosage of 0.24 mg/kg (injected subcutaneously) is recommended for hematopoietic stem cell mobilization and subsequent autologous transplantation. Thus, our results indicate that in order for a synergistic effect to occur, it might be necessary to raise the concentration of plerixafor used in a clinical setting, receiving higher concentrations than those measured in the blood of individuals treated with plerixafor. However, many factors are at play in the more complex clinical setting and could influence drug interaction and, thus, concentration needed for synergistic effect.

In the present study, we observed that concomitant administration of plerixafor and rituximab is of great importance (Fig. 1); an observation especially relevant if plerixafor is to be tested for inclusion into the DLBCL treatment regimen. In support of this observation, Kozin *et al.* [40] demonstrated that concomitant treatment was superior to sequential drug administration, showing that treatment with plerixafor immediately after local irradiation in breast and lung carcinoma murine xenograft models resulted in significant inhibition of tumor re-growth, whereas administration of plerixafor 5 days post-irradiation treatment rendered plerixafor without effect.

When exploring the mechanism of drug effect, we observed that the fraction of apoptotic cells increased upon combined treatment with rituximab and plerixafor (Fig. 3). This implies that apoptosis induction is a contributing factor to the enhanced effect observed when subjecting DLBCL cells to plerixafor in combination with rituximab. In accordance, Beider *et al.* [34] observed the CXCR4 antagonist BKT140 to reverse rituximab-induced cellular arrest of Burkitt lymphoma cells, demonstrating that the number of late apoptotic/dead cells increased significantly upon combination treatment, with increased activation of the apoptotic caspase3 pathway.

The therapeutic effect of plerixafor is likely two-fold; in addition to its direct sensitizing properties, plerixafor might render CXCR4-expressing tumor cells more accessible to rituximab-induced eradication by inhibiting tumor cell homing to the bone marrow and by mobilizing tumor cells to the peripheral blood [41, 42]. In line with this, Beider *et al.* [34] observed that the survival and proliferation of CXCR4-expressing tumor cells were supported by the stroma and that this bone marrow stromal cell-induced protection of Burkitt lymphoma cells could be reversed by the CXCR4 antagonist BKT140, increasing rituximab-induced tumor cell death. Accordingly, Buchner *et al.* [43] demonstrated that CXCR4 antagonists abrogate the stromal cell-induced protection of CLL cells and, thereby, increase the efficacy of rituximab-induced CDC.

Recently, it was reported that non-Hodgkin lymphoma patients experiencing a decrease in bone marrow-expressed CXCR4 after treatment, responded well and had a significantly better prognosis [32]. Hence, we investigated the drug-induced effect on CXCR4 surface expression level, and found plerixafor to induce a decrease in CXCR4 fluorescence intensity, as measured by flow cytometry (Fig. 4). This could either indicate plerixafor-induced internalization of CXCR4 or masking of the receptor due to plerixafor binding; but in either case, it indicates that plerixafor decreases CXCR4 receptor accessibility. Consequently, our observation that combined treatment is more effective in the RIVA cell line might not only be due to the higher rituximab sensitivity of this cell line, but also that more plerixafor molecules have bound to RIVA cells and/or that plerixafor induces a greater downregulation of CXCR4 on the surface of RIVA cells. Kim *et al.* [44] and Moreno *et al.* [18] suggest plerixafor treatment to cause internalization of myeloma and DLBCL cell surface-located CXCR4, respectively, as assessed by flow cytometry; whereas Fricker *et al.* [45] used flow cytometry-based assessment of anti-CXCR4 antibody binding as an indirect measure for binding of plerixafor to CXCR4. Also using flow cytometry, Schols *et al.* [46] more thoroughly investigated the interaction between plerixafor, anti-CXCR4 antibody, and the CXCR4 receptor, in T-cell lymphoblastic lymphoma cells. They report that binding of plerixafor to the CXCR4 receptor inhibits anti-CXCR4 antibody binding and declare that CXCR4 internalization does not occur, which they later confirmed by fluorescence microscopy-based visualization using stably transfected U87.CD4 cells expressing GFP-coupled CXCR4 [47]. Hence, the plerixafor-induced decrease in CXCR4 fluorescence intensity observed in our study is most likely a measure of receptor-bound plerixafor and not due to receptor internalization. Of notice, Beider *et al.* [34] observed that Burkitt lymphoma cell interaction with bone marrow stromal cells increased the tumor cell surface expression of CXCR4. Thus, the effect of plerixafor on the amount of available tumor cell CXCR4 receptors is probably both direct via receptor blockade and indirect via disruption of tumor-stroma cell interaction, preventing the stroma-induced increase in CXCR4 expression.

Patients diagnosed with ABC-DLBCL have a dismal prognosis [48]. A frequent characteristic of ABC-DLBCL is constitutive activation of the NF-kB signaling pathway due to gain/loss-of-function mutations in genes encoding pathway members upstream of the central transcription factor NF-kB [49]. Of notice, NF-kB can induce transcription of CXCR4, and CXCR4 pathway activity can result in nuclear accumulation of NF-kB [50, 51]. Accordingly, Chen *et al.* discovered an association between high CXCR4 expression level and ABC subtype [19], while Shin *et al.* reports high NF-kB expression in DLBCL samples to associate with CXCR4 expression [52]. By disrupting the positive feedback loop between CXCR4 and NF-kB, the CXCR4 antagonist plerixafor could possibly counterbalance the NF-kB signaling pathway deregulation. In support, Huang *et al.* observed that pre-treatment of osteosarcoma cells with plerixafor attenuated the CXCL12-induced increase in NF-kB promoter activity [53], with similar results reported for oral squamous cell carcinoma [54].

Like the ABC-DLBCL-diagnosed patients, double-hit lymphoma patients have a poor outcome [55]. Most commonly, double-hit lymphoma cases are of the GCB subtype and characterized by concurrent chromosomal translocations of *MYC* and *BCL2*, resulting in deregulation of these oncogenes and, consequently, in deregulation of the cell cycle process and apoptosis [56, 57]. Cancer cells of patients diagnosed with GCB-DLBCL are frequently addicted to PI3K/Akt signaling, and degradation of Myc can be inhibited as a consequence of Akt-induced inactivation of GSK-3β, whereas sequestering of Bcl-2 can be disrupted by Akt-induced phosphorylation of the proapoptotic protein Bad [49, 58, 59]. Since the PI3K/Akt signaling pathway is central to CXCR4 signaling [51], plerixafor could possibly attenuate the translocation-induced deregulation of *MYC* and *BCL2*. In agreement, Chen *et al.* observed an association between CXCR4-positivity of DLBCL tumor samples and high expression of *MYC* as well as *BCL2* [19]; whereas Hatano *et al* [60] demonstrated decreased c-Myc expression and Mao *et al.* [61] decreased Bcl-2 expression upon plerixafor administration, though in prostate cancer cells and brain tissue, respectively. Thus, plerixafor additionally has potential as adjuvant therapy for double-hit lymphoma patients.

## Conclusions

Based on the promising findings of this study, implying that the CXCR4 antagonist plerixafor synergistically enhances the anti-proliferative/pro-apoptotic effect of rituximab on DLBCL cells, it seems interesting to further explore the effect of combining rituximab with CXCR4 antagonism. If our findings are reproducible in a clinical setting, adding plerixafor to the R-CHOP regimen could result in reduced development of drug resistance and, ultimately, in improved patient survival; by combining synergistically interacting drugs, the therapeutic effect might be either increased, or maintained while lowering drug-induced toxicity by administering lower doses of the drugs [29].

## Abbreviations

7-AAD, 7-Amino-Actinomycin; ALL, acute lymphoblastic leukemia; CDC, complement-dependent cytotoxicity; CLL, chronic lymphocytic leukemia; CXCL12, C-X-C chemokine receptor type 4; CXCR4, C-X-C chemokine ligand type 12; DLBCL, diffuse large B-cell lymphoma; HS, Pooled Human AB Serum

## Additional files

Additional file 1: Systematic dose-response experiments for rituximab. The diffuse large B-cell lymphoma (DLBCL) cell lines RIVA and FARAGE were exposed to a two-fold serial dilution of the anti-CD20 antibody rituximab (15 different concentrations starting from 66.67 μg/mL) for 0 h and 48 h and, subsequently, incubated 2 h with the MTS-containing CellTiter 96® AQ_ueous_ One Solution Reagent (Promega) before absorbance measurements at 492 nm. Thus, the center of MTS exposure was at 1 h and 49 h, respectively. Model-based pre-processing was performed using raw absorbance values obtained through these MTS-based experiments, and the *G*-model used to generate dose-response curves and obtain time-independent summary statistics, as previously described [27]. Dose-response experiments were repeated thrice for each cell line, using a minimum of three technical replicates per condition. (PDF 5 kb) Additional file 2: Data used to generate Figure S1. (XLSX 26 kb) Additional file 3: Data used to generate Figure S2. (XLSX 18 kb) Additional file 4: Data used to generate Figure S3. (XLSX 16 kb) Additional file 5: Data used to generate Figure S4. (XLSX 16 kb)
