# Supplementary figures and images for: The CXCR4 antagonist plerixafor enhances the effect of rituximab in diffuse large B-cell lymphoma cell lines
Source: Biomark Res. 2016 Jun 14;4:12. doi: 10.1186/s40364-016-0067-2 (PMC4908729; doi:10.1186/s40364-016-0067-2)

Rituximab

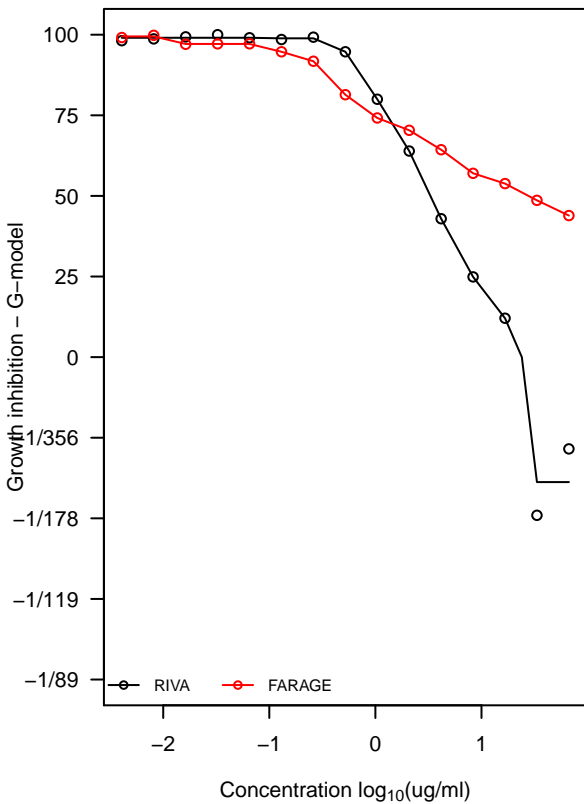Rituximab induced  $\text{GI}_{50}^{\text{G}}$ 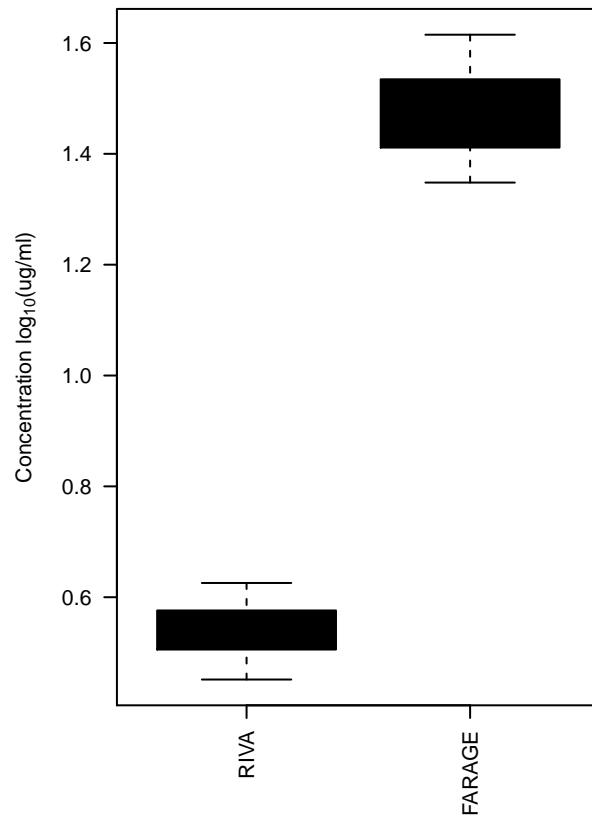Rituximab induced  $\text{AUC}_0^{\text{G}}$ 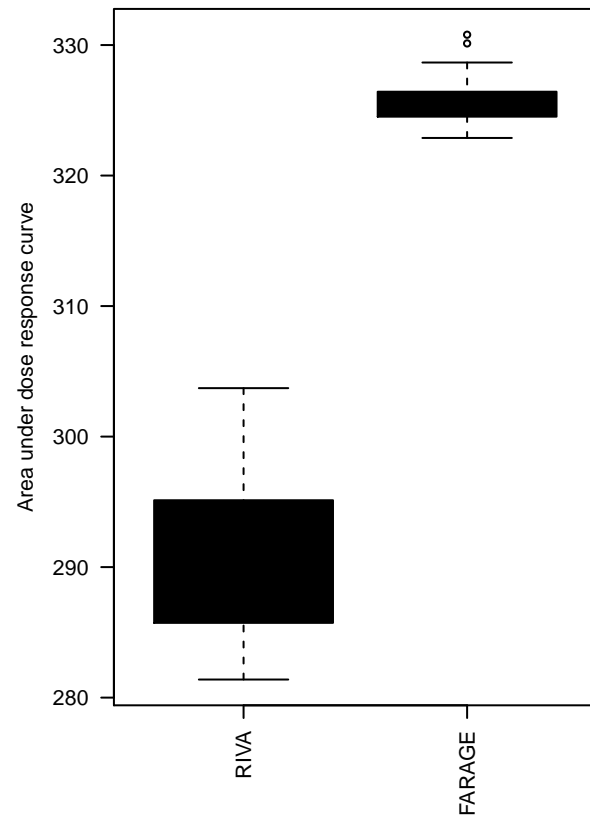

Supplement: Additional file 1: — Systematic dose-response experiments for rituximab. The diffuse large B-cell lymphoma (DLBCL) cell lines RIVA and FARAGE were exposed to a two-fold serial dilution of the anti-CD20 antibody rituximab (15 different concentrations starting from 66.67 μg/mL) for 0 h and 48 h and, subsequently, incubated 2 h with the MTS-containing CellTiter 96® AQueous One Solution Reagent (Promega) before absorbance measurements at 492 nm. Thus, the center of MTS exposure was at 1 h and 49 h, respectively. Model-based pre-processing was performed using raw absorbance values obtained through these MTS-based experiments, and the G-model used to generate dose-response curves and obtain time-independent summary statistics, as previously described [27]. Dose-response experiments were repeated thrice for each cell line, using a minimum of three technical replicates per condition. (PDF 5 kb) [file 40364_2016_67_MOESM1_ESM.pdf]
